# BINGE EATING DISORDER AND QUALITY OF LIFE OF CANDIDATES TO BARIATRIC SURGERY

**DOI:** 10.1590/S0102-6720201500S100015

**Published:** 2015-12

**Authors:** Ana Júlia Rosa Barcelos COSTA, Sônia Lopes PINTO

**Affiliations:** Bariatric Outpatient Unit, Federal University of Tocantins, Palmas, TO, Brazil

**Keywords:** Binge eating disorder, Quality of Life, Bariatric Surgery

## Abstract

***Background*:**

Obesity decreases the quality of life, which is aggravated by the association of comorbidities, and the binge eating disorder is directly related to body image and predisposes to overweight.

**Aim:**

Evaluate association between the presence and the level of binge eating disorder and the quality of life of the obese candidates for bariatric surgery.

***Methods*:**

Cross-sectional study analyzing anthropometric data (weight and height) and socioeconomics (age, sex, marital status, education and income). The application of Binge Eating Scale was held for diagnosis of Binge Eating Disorder and the Medical Outcomes Study 36-item Short-From Health Survey to assess the quality of life.

***Results*:**

Total sample studied was 96 patients, mean age 38.15±9.6 years, 80.2% female, 67.7% married, 41% with complete and incomplete higher education, 77.1% with lower income or equal to four the minimum salary, 59.3% with grade III obesity. Binge eating disorder was observed in 44.2% of patients (29.9% moderate and 14.3% severe), and these had the worst scores in all domains of quality of life SF36 scale; however, this difference was not statistically significant. Only the nutritional status presented significant statistically association with the presence of binge eating disorder.

***Conclusion*:**

High prevalence of patients with binge eating disorder was found and they presented the worst scores in all domains of quality of life.

## Abstract (pt)

***Racional*:**

A obesidade diminui a qualidade de vida que se agrava com a associação de comorbidades. O transtorno da compulsão alimentar tem relação direta com a imagem corporal e predispõe ao excesso de peso.

***Objetivo*:**

Avaliar a associação entre a presença e o nível do transtorno da compulsão alimentar periódica e a qualidade de vida dos pacientes obesos candidatos à cirurgia bariátrica.

***Métodos*:**

Estudo ambulatorial do tipo transversal onde coletaram-se dados antropométricos (peso e altura) e socioeconômicos (idade, sexo, estado civil, escolaridade e renda). Realizou-se aplicação da Escala de Compulsão Alimentar Periódica para diagnóstico do Transtorno de Compulsão Alimentar Periódica - TCAP e o Medical Outcomes Study 36-item Short-From Health Survey para avaliar a qualidade de vida.

***Resultados*:**

Amostra total estudada foi de 96 pacientes, idade média de 38,15±9,6 anos, 80,2% do sexo feminino, 67,7% casados, 41% com ensino superior completo/incompleto, 77,1% com renda ≤4 salários mínimos, 59,3% obesidade grau III. Observou-se transtorno da compulsão alimentar em 44,2% dos pacientes (29,9% moderado e 14,3% grave), e estes apresentavam os piores escores em todos os domínios de qualidade de vida da escala SF36; porém, esta diferença não foi estatisticamente significante. Somente o estado nutricional apresentou associação com a presença do transtorno.

***Conclusão*:**

Constatou-se

**e:**

levada prevalência de pacientes com transtorno da compulsão alimentar e eles apresentaram os piores escores em todos os domínios da qualidade de vida.

## INTRODUCTION

Obesity is neither a transmissible disease nor is it an injury; it is progressive and recurrent, and expressed as the accumulation of fat or energy as triglycerides in the adipose tissue, resulting in increased body weight, exceeding 15% or more of the optimum weight^9^^,^ 21. It is a neurochemical disorder causing changes in the body composition. It is regarded as pandemic as it results from the interaction of biological, sociodemographic and behavioral factors. This is evident from four inquiries already performed in Brazil, in both genders, viz., the National Study of Family Expenses (ENDEF) (1974-1975)^13^, National Health and Nutrition Survey (PNSN) (1989)^14^ and the Family Budget Research (POF) and POF 2008-200916 2002-2003^15^. Between 1974 and 2009 the prevalence of overweight adults soared to almost three times in men (from 18.5% to 50.1%) and nearly twice in women (from 28.7% to 48.0%). At the same time, obesity increased by more than four-fold in men, from 2.8% to 12.4% and two-fold in women, from 8.0% to 16.9%^13^^,^ 14 ^,^ 15 ^,^ 16. This reality is visible across all the regions of Brazil and is steadily increasing up to approximately the 45-54 years old group in men and the 55-64 age segment in women; after this point, the accumulation of excess weight tends to decrease^16^.

Morbid obesity, body mass index greater than or equal to 40 kg/m², increased by 255% in Brazil, according to the ENDEF, PNSN and POF and, in the North between 1974 and 2003, there was an increase of 410%^16^.

Obesity directly affects the quality of life, precipitating changes in the body image of a person due to excess weight, causing them to feel undervalued^24^. Costa et al.,^6^ observed a clear relationship between body image and binge-eating episodes in morbidly obese patients, in which the higher the frequency of binge eating the greater the concern regarding body image^16^.

Binge Eating Disorder (BED / DSM IV-TR) is a syndrome involving the loss of control over the type and quantity of food consumed at least twice a week and not accompanied by compensatory behavior^1^. It is a psychopathological condition which leads to obesity early on and, more seriously, hinders the response to dietary treatment. Therefore, it necessitates greater length of time on diets^2^^,^ 19.

Due to the difficulty in achieving success after weight reduction programs, bariatric surgery has increased in popularity, and is being performed with great success. However, the concern regarding behavioral changes as a consequence of the surgery continues to remain a risk factor.

Therefore, the objective of the current study is to evaluate the association between the presence and the degree of Binge Eating Disorder, and investigate the level of the Quality of Life of the obese patients who are candidates for bariatric surgery.

## METHODS

This is a cross-sectional study including all the patient candidates for bariatric surgery who sought care between November 2013 and February 2015 at the Bariatric Clinic - AMBBAR, Federal University of Tocantins, Palmas, TO, Brazil. This project was approved of by the Research Ethics Committee of the University, 039/2014 process. All the patients signed the Informed Consent and Informed acceptance of participation.

### Data collection

Sociodemographic information, as well as data on nutritional status, binge eating and quality of life were collected.

Sociodemographic data were taken from the completed medical records after consultation with the patient. This included information concerning the age in years, gender, marital status, family income in minimum wages and education.

To evaluate the nutritional status the anthropometric measurements of weight and height were ascertained. Body weight was recorded after weighing on an electronic digital scale with a maximum capacity of 300 kg, and an accuracy of 0.05 g. To measure the height was used a graduated stadiometer in accordance with Lohman technique^18^.

The Body Mass Index (BMI) was calculated from the weight measured in kg/m² based on the recommendations of the Brazilian Society for Bariatric and Metabolic Surgery (2008).

### Binge eating

To evaluate the possible presence of Binge Eating Disorder (BED) was used the Binge Eating Scale developed by Gormally et al.^11^ and validated by Freitas et al.^10^. This involves a self-administered questionnaire, which includes 16 items and 62 statements. For each item only one statement, which best represents the individual's response, must be selected. Each statement has a score, with "0" indicating absence and "3" implying the maximum severity of BED. Finally, the points of the statements selected are added. If the final scores presented are lower than or equal to 17, the patient is considered to be without BED; scores between 18 and 26 imply those with moderate compulsion; scores greater than or equal to 27 indicate serious or grave compulsion^11^.

### Quality of life

Preoperatively, was utilized the evaluation questionnaire, the final version of which had been developed and released in 1993 by John Ware and his team from the Medical Outcomes Study 36-Item Short-Form Health Survey (SF-36). This had been validated by Ciconelli in 1999 in Brazil^4^. It includes 36 items divided into eight sections that help to detect the patient's state of health. It is divided into the physical (functional capacity, physical aspects, pain and general health) and mental components (vitality, social functioning, emotional aspects and mental health)^4^. It gives both a general assessment and subjective self-perception, estimating both the positive and negative points^3^. It is a self-administered questionnaire which the patient completes post consultation. The score percentage is done on a scale of 0 to 100, and the higher the score, the better the perceived quality of life^25^.

### Statistical analysis

Database was constructed using Excel 2013 for Windows program and the analysis was done with the SPSS software version 20.0. To assess the normality of the continuous variables was used the Kolmogorov-Smirnov test and produced the histogram. When there was a normal distribution was performed Student t-test whereas for the other variables the Mann-Whitney test. Was used the chi-square test to assess the categorical variables. To measure the association between the variables, the level of statistical significance p<0.05 was considered.

## RESULTS

This sample included 96 obese patients with a mean age of 38.15±9.6 years, a minimum of 20 years and a maximum of 69. Among them 80.2% were women (n=77), 67.7% (n=65) were married, 41% (n=39) with complete/incomplete higher education, and 77.1% (n=74) reported an income of less than four minimum salaries. Regarding their nutritional status, it was observed that 59.3% (n=57) were classified as having grade III obesity and 32.3% (n=31) were superobese, confirming the indication for bariatric surgery (Table 1).

**TABLE 1:** - Demographic and anthropometric data of obese bariatric surgery candidates (n=96)

**Variable**	**n**	**%**
Gender		
Female	77	80.2
Male	19	19.8
Marital status		
Single / Widowed	31	32.3
Married / Unwed but living together	65	67.7
Education		
Elementary school (in) complete	18	17.9
High school (in) complete	38	40
Higher education (in) complete	39	41
Income		
<4 SM*	74	77.1
>4 SM*	22	22.9
Nutritional status		
Obese grade I	2	2.1
Obese grade II	6	6.3
Obese grade III	57	59.3
Superobese	31	32.3

* SM=minimum wages

**FIGURE 1:**
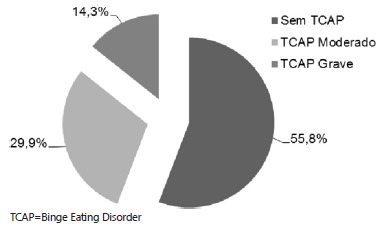
- Binge eating according to the Binge Eating Scale - ECAP obese candidates for bariatric surgery (n=77)

Among the 96 patients treated at the clinic, 77 (80%) completed the Binge Eating Scale questionnaire. The final results revealed that 44.2% had Binge Eating Disorder (BED/TCAP), 29.9% of them to a moderate level and 14.3% to a severe degree (Figure 1).

TCAP=Binge Eating Disorder

Regarding the patients with and without BED, no statistically significant relationship was observed among gender, marital status, education and income. Regarding the nutritional status, however, there was a significant difference between the groups with and without BED, having grades I, II and III obesity associated with binge eating (p=0.05, Table 2).

**TABLE 2:** - Demographic and anthropometric data of obese patients based on the presence or absence of BED (n=77)

**Variable**	**Group with TCAP (n=34)**	**Group without TCAP (n=43)**	p*
Gender			
Female	28	33	0.54
Male	6	10	
Marital status			
Single / Widowed	11	12	0.67
Married / Unwed but living together	23	31	
Education			
Elementary school (in) complete	9	5	0.23
High school (in) complete	13	17	
Higher education (in) complete	20	12	
Income			
<4 SM**	28	34	0.71
>4 SM	6	9	
Nutritional status			
Obese class I, II, III	18	32	0.05
superobese	16	11	

* Chi-square test; ** SM=minimum wage; TCAP=binge eating disorder

Regarding the quality of life, the worst scores were observed in the categories of Functional Capacity, Physical Limitations and Pain. By contrast the domain with the best result was Mental Health (Table 3).

Among the 96 patients treated only 36 (37.5%) completed both questionnaires (Binge Eating Scale Periodic and SF-36). The distribution in the Quality of Life domain observed among the patients with and without BED showed that the group had the worst scores of quality of life; however, this difference was not statistically significant (Table 3).

**TABLE 3:** - Evaluation of the areas of the SF-36 scale of obese patients based on the presence or absence of BED (n=36)

**Domains**	With BED Average or median*	Without BED Average or median*	**Mean total**	**p**
Functional capacity	40.1	53.7	48.8	0.09^2^
Physical limitations	25.0	50.0	44.5	0.53^1^
Aches	43.6	44.1	43,9	0.94^2^
General health	49.0	53.8	52.1	0.51^2^
Vitality	52.6	53.4	53.1	0.91^2^
Social aspects	51.9	65.3	60.4	0.18^2^
Emotional aspects	33.3	66.7	50.4	0.46^1^
Mental health	57.2	67.4	63.7	0.24^2^

*For variables with normal distribution, was used the average, and no normality as the median; (1)=Mann-Whitney test; (2)=t-test

## DISCUSSION

Patients with psychiatric problems - in particular mood, anxiety and psychotic disorders - are commonly considered contraindicated for bariatric surgery. However, no real accurate data or predictors of good or poor prognosis or even adequate and/or proven studies are available^23^. Nevertheless, it is known that very restrictive operations and poorly absorptive procedures, such as gastrojejunal bypass and Roux-en-Y gastric bypass, in general do not show good results in compulsive patients. This is because they do not follow the limitations with regard to the amount of food ingested, which can result in postoperative complications, as well as become an important factor for the regained weight. This will ultimately lead the patient to undergo a new operation/depression and/or death^8^.

Diaz et al.^7^ after surveying 45 obese persons who underwent bariatric surgery, observed that, those with binge eating tendencies, suffered from more complications with a lower improvement resolution rate in hypertension.

In the study by Hsu et al.^12^ conducted on 37 adults awaiting gastric bypass surgery, TCAP prevalence was observed in 25%, 11% of which were severe and 14% moderate. Zanella^26^, already involved in a study of 50 morbidly obese patients, found that 36% had TCAP. Brimann et al.^3^ in their study on 73 patients reported 35.6% with severe obesity and 13.7% with moderate; the study also showed that the greater the obesity, the lesser the quality of life^3^.

In the present study, the prevalence of BED was observed in 44.2% of the study population; this is a high result when compared with the literature. In the case of a severely obese population predominantly the result was expected, because these were individuals with limited control of caloric intake.

Correlating obesity, binge eating disorder and quality of life, Petribú et al.^20^ conducted a study utilizing 67 patients in the preoperative state. They detected a prevalence of 56% of patients with BED having the worst quality of life scores and showing significant differences in all the domains. These results reveal that obesity interferes with the individual's quality of life in various aspects including the physical, emotional, psychological and social.

Although this study did not present any association between BED and the quality of life, it was observed that patients with BED had lower scores in all domains on the SF-36 questionnaire. From these results it can be understood that obesity presents a trend in a reduced quality of life of these individuals when compared with the non-compulsive individuals; however, the difference was not statistically significant, probably due the limitations imposed by sample size.

## CONCLUSION

A high prevalence of patients with binge-eating disorder was identified, and they revealed the worst scores in all the quality of life domains.
